# Insight into the current genomic diversity, conservation status and population structure of Tunisian Barbarine sheep breed

**DOI:** 10.3389/fgene.2024.1379086

**Published:** 2024-05-31

**Authors:** Samira Giovannini, Giorgio Chessari, Silvia Riggio, Donata Marletta, Maria Teresa Sardina, Salvatore Mastrangelo, Francesca Maria Sarti

**Affiliations:** ^1^ Dipartimento di Scienze Agrarie, Alimentari e Ambientali, University of Perugia, Perugia, Italy; ^2^ Dipartimento Agricoltura, Alimentazione e Ambiente, University of Catania, Catania, Italy; ^3^ Dipartimento Scienze Agrarie, Alimentari e Forestali, University of Palermo, Palermo, Italy

**Keywords:** local sheep breed, single nucleotide polymorphism, genetic differentiation analyses, livestock conservation, Africa, fat-tail

## Abstract

Local livestock breeds play a crucial role in global biodiversity, connecting natural and human-influenced environments and contributing significantly to ecosystem services. While commercial breeds dominate industrial systems, local livestock breeds in developing countries, like Barbarine sheep in Tunisia, are vital for food security and community maintenance. The Tunisian Barbarine sheep, known for its adaptability and distinctive fat-tailed morphology, faces challenges due to historical crossbreeding. In this study, the Illumina Ovine SNP50K BeadChip array was used to perform a genome-wide characterization of Tunisian Barbarine sheep to investigate its genetic diversity, the genome structure, and the relationship within the context of Mediterranean breeds. The results show moderate genetic diversity and low inbreeding. Runs of Homozygosity analysis find genomic regions linked to important traits, including fat tail characteristics. Genomic relationship analysis shows proximity to Algerian thin-tailed breeds, suggesting crossbreeding impacts. Admixture analysis reveals unique genetic patterns, emphasizing the Tunisian Barbarine’s identity within the Mediterranean context and its closeness to African breeds. Current results represent a starting point for the creation of monitoring and conservation plans. In summary, despite genetic dilution due to crossbreeding, the identification of genomic regions offers crucial insights for conservation. The study confirms the importance of preserving unique genetic characteristics of local breeds, particularly in the face of ongoing crossbreeding practices and environmental challenges. These findings contribute valuable insights for the sustainable management of this unique genetic reservoir, supporting local economies and preserving sheep species biodiversity.

## 1 Introduction

Local breeds of domesticated livestock species play a pivotal role in global biodiversity, acting as a crucial link between natural and human-influenced environments and contributing significantly to ecosystem services (Sponenberg et al., 2019). Despite their implications as an important part of the cultural and genetic heritage, the Domestic Animal Diversity Information System (DAD-IS) reports that out of the 7,745 existing local livestock breeds, 23.77% are at risk of extinction, with 1,841 classified as “at risk” and 4,439 as “at unknown risk” (https://www.fao.org/dad-is/, accessed 10/01/2024). The primary purpose of domestic animals lies in their essential role in food production, particularly through the significant contribution of commercial breeds in industrial systems found in both developed and emerging countries. However, in rural areas of developing countries, local livestock breeds play a vital role in ensuring food security, nutrition, and health (Leroy et al., 2018).

Tunisian Barbarine sheep is an example of local breed and occupies a prominent position within the livestock diversity of Tunisia. Notably recognized for its adaptability to arid environments and distinctive fat-tailed morphology, Tunisian Barbarine stands out among fat-tailed sheep breeds widespread in North Africa, particularly in Tunisia, Libya, and Algeria (Khaldi et al., 2011). Tunisian Barbarine is a medium-sized meat-type sheep characterized by creamy wool, with red or black-colored faces and legs; this breed has historically undergone crossbreeding with thin-tailed breeds driven by consumer demand and taste preferences (Ben Salem et al., 2011). Once the historical appreciation for fat production ended, this practice of crossbreeding began, posing challenges to preserve the breed’s unique genetic traits. Additionally, the breed demands extra labor for reproduction: the presence of a fat tail inhibits natural mating, necessitating the assistance of shepherds to lift it during copulation. This has also contributed to the preference shift towards thin-tailed breeds (Bedhiaf-Romdhani et al., 2008). Believed to be the progenitor of the “Tunis” sheep breed in the United States (Gammoudi and Bedhiaf, 2015) and, potentially influencing the Italian Barbaresca breed (Tolone et al., 2012), the Tunisian Barbarine breed plays a crucial role in the livelihoods of Tunisian communities. Its resilience in challenging arid climatic conditions stems from its ability to deposit and mobilize body reserves, primarily from the tail and the rest of the body (Atti et al., 2004; Ben Salem et al., 2011).

In the present worldwide scenario, where the significance of local livestock is pivotal for both food security and livelihoods, the importance of genetic diversity and distinct phenotypic traits cannot be ignored. Managing animal genetic resources requires a comprehensive study of genetic implications, such as genetic variability and population structure (FAO, 2007; Scherf and Pilling, 2015), to avoid diminished fitness, decreased productivity, and the potential risk of extinction (Keller and Waller, 2002; Charlesworth and Charlesworth, 2010). In recent years, advances in genetic research with molecular markers have facilitated the exploration of genetic characteristics in various species and breeds, highlighting economically important traits (Tolone et al., 2012; Scherf and Pilling, 2015; Yuan et al., 2017). These studies have employed diverse analytical strategies, including the investigation of genetic diversity indices, examination of allele frequencies, construction of phylogenetic trees and exploration of genomic relationships between breeds (Kizilaslan et al., 2023). Several genome-wide SNP studies applied on Tunisian Barbarine have already described its genetic structure (Belabdi et al., 2019; Bedhiaf-Romdhani et al., 2020; Baazaoui et al., 2021; Baazaoui et al., 2024). Despite being close to other thin-tailed breeds of the Maghreb region, Tunisian Barbarine sheep exhibit a stronger genetic affinity with South-Eastern Mediterranean breeds (Baazaoui et al., 2024). Extensive gene flow among Tunisian sheep breeds, combined with uncontrolled reproductive management and the lack of breed development programs, has resulted in widespread crossbreeding in favor of thin-tailed breeds hindering substantial genetic homogenization (Bedhiaf-Romdhani et al., 2020). However, the Tunisian Barbarine breed retains a unique genetic identity, with candidate genes like *BMP2* associated with lipid storage, reflecting selective pressures and adaptation to local conditions (Belabdi et al., 2019; Baazaoui et al., 2021; Baazaoui et al., 2024). In this research, we expanded the scope by including evaluations of other Barbarine samples from different geographic regions. This approach allows for a more comprehensive understanding and comparison of genetic diversity, population dynamics, and unique traits within the Tunisian Barbarine breed. Through the inclusion of multiple Barbarine populations, the aim is to provide a more nuanced perspective on the genetic structure and conservation status of this unique genetic reservoir for sustainable management.

## 2 Materials and methods

### 2.1 Sampling and genotyping

**FIGURE 1:**
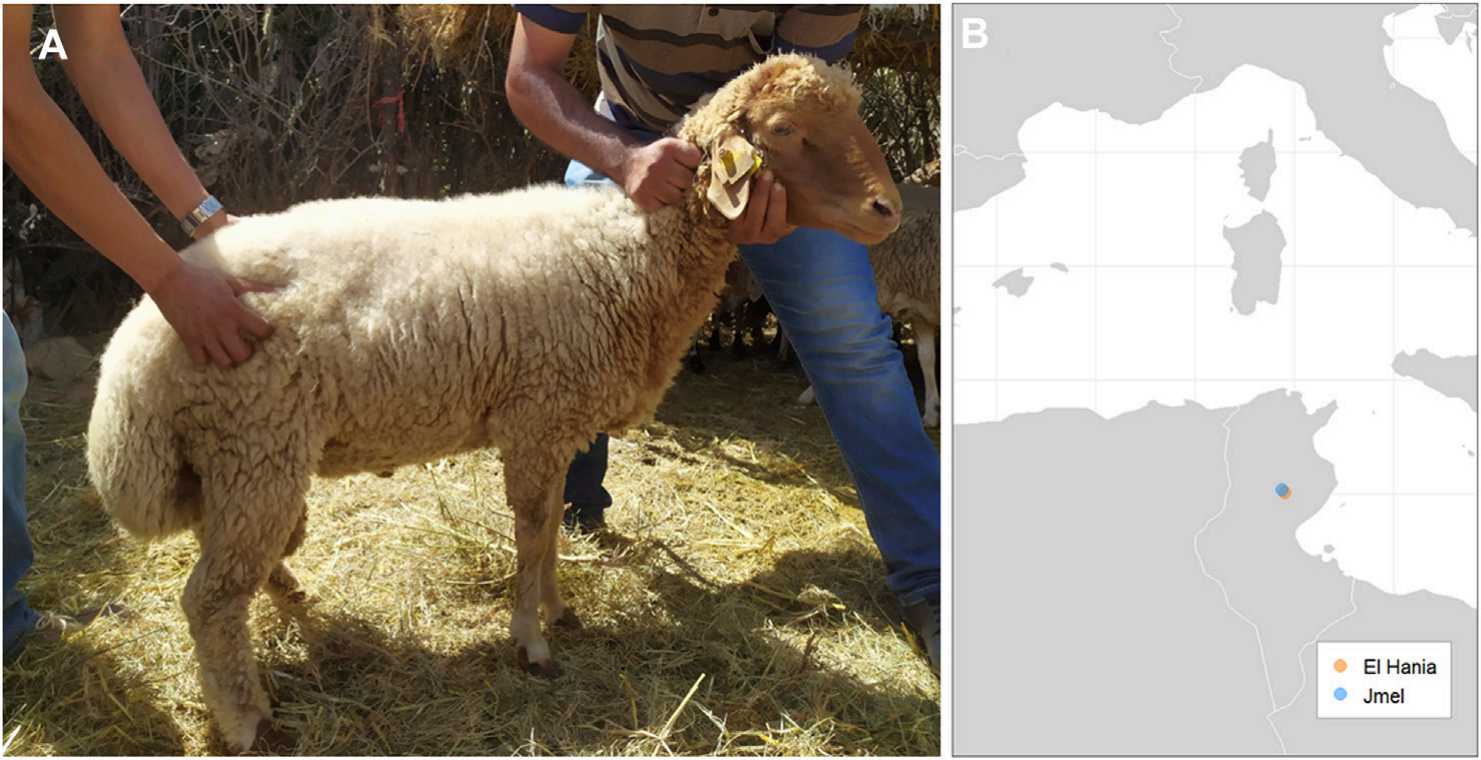
**(A)** Picture of a Tunisian Barbarine sheep sampled. **(B)** Area of sampling (Jmel and El Hania, Sidi Bouzid, Tunisia).

A total of 24 Tunisian Barbarine sheep blood samples (BARB dataset) were obtained from selected farms that were part of a Cooperation project (2018–2022) led by Tamat (Italian NGO https://tamat.org) (Figure 1). The animals were selected among the families of breeders based on phenotypic features and information provided by farmers to ensure the collection of unrelated individuals. DNA extraction from blood was performed using the Illustra blood genomic Prep Mini Spin kit (GE Healthcare, Little Chalfont, United Kingdom). The samples were genotyped using the Illumina OvineSNP50 BeadChip, resulting in a total of 53,516 SNPs (Illumina, Inc., San Diego, CA, United States).

### 2.2 Data management and quality control

PLINK ver. 1.9 (Chang et al., 2015) was used to filter data and perform the quality control. Chromosomal coordinates of SNPs were referred to the OAR4.0 genome assembly (NCBI accession number GCA_000298735.2). Markers on unknown and sexual chromosomes were removed. The quality parameters applied were the following: a minor allele frequency ≥0.05, a genotype call rate for a SNP ≥0.95 and an individual call rate ≥0.99, resulting in 43,630 variants and 24 sheep (BARB dataset). To analyze the breed in a Mediterranean context, the raw data of Tunisian Barbarine were combined with genotypic data of 59 sheep breeds genotyped with Illumina Ovine SNP50K BeadChip array (hereafter called MED_POP dataset) (Kijas et al., 2012; Ciani et al., 2014; Ciani et al., 2015; Manunza et al., 2016; Gaouar et al., 2017; Mastrangelo et al., 2017; Mwacharo et al., 2017; Mastrangelo et al., 2018; Ben Jemaa et al., 2019; Ciani et al., 2020; Chessari et al., 2023). The random sampling selection procedure implemented in the BITE R package (Milanesi et al., 2017) was used to select a maximum of 30 representative individuals per breed. PLINK ver. 1.9 (Chang et al., 2015) was also used to perform quality control on MED_POP dataset: a minor allele frequency ≥0.05, a genotype call rate for a SNP ≥0.95 and an individual call rate ≥0.99. Furthermore, markers with high linkage disequilibrium (LD) were removed by applying the function--indep-pairwise with an LD threshold of *r* ^2^ > 0.2, a window size of 50 SNPs and a window step size of 10 SNPs. The filtered dataset contained a total of 33,049 variants, 60 breeds and 1,151 animals, from 10 countries: Albania, Algeria, Cyprus, Egypt, Greece, Israel, Italy, Libya, Spain, Tunisia. The breeds of the final dataset used for the population analyses are summarized in Supplementary Table S1.

### 2.3 Genetic diversity and run of homozygosity (ROH) analyses

BARB dataset was examined through PLINK ver. 1.9 (Chang et al., 2015) to estimate observed (H_O_) and expected (H_E_) heterozygosity, the inbreeding coefficient (F_IS_) and the average minor allele frequency (MAF). Additionally, historical trends in effective population size (N_e_) were assessed using SNeP v1.1 software (Barbato et al., 2015), while contemporary N_e_ was also determined using NeEstimator ver. 2.1 (Do et al., 2014), following the random mating option, within the linkage disequilibrium method proposed by Waples and Do (Waples and Do, 2010).

The analysis of Runs of Homozygosity (ROH) was conducted using the consecutive runs method implemented in the R package detectRUNS (Biscarini et al., 2018). Specific parameters were defined for the inclusion of a ROH: (i) a minimum of 15 SNPs, (ii) no opposite genotypes (maxOppRun = 0), (iii) no missing genotypes within a ROH (maxMissRun = 0), (iv) the maximum allowed gap between consecutive SNPs within a ROH was set at 250 kb, and (v) the minimum length of a ROH was established at 1 Mb.

ROH segments were categorized into five length classes following the nomenclature in literature (Kirin et al., 2010; Ferenčaković et al., 2013): 1–2, 2–4, 4–8, 8–16, and >16 Mbp. The mean number of ROH per individual (N_ROH_) and chromosome (NC_ROH_), as well as the average length of ROH in Mb per individual (L_ROH_) and chromosome (LC_ROH_) were computed. Furthermore, the genomic inbreeding coefficient (F_ROH_) was calculated by the total length of the genome comprised in the ROH for each individual divided by the total autosomal genome length (∼2.4 Gb).

The identification of markers present in the rich homozygous regions (ROH islands) was performed through the computation of the standard normal z-score from the incidence of all SNPs within ROHs and deriving *p*-values. The markers within the top 0.1% were considered to constitute ROH islands. The genomic coordinates of markers were examined using NCBI Genome Data Viewer according to Assembly OAR4.0 (GCA_000298735.2). QTL information from the Animal QTL Database for the OAR4.0 assembly, release 48, was used.

### 2.4 Population genetic analysis and structure

Population relationships and structure were studied using merged datasets (MED_POP dataset).

To explore individual similarities within the matrix, multidimensional scaling (MDS) analysis on pairwise identity-by-state (IBS) distances was performed using PLINK ver. 1.9 (Chang et al., 2015), with the—cluster and—mds-plot functions.

Additionally, Arlequin ver. 3.5.2.2 (Excoffier and Lischer, 2010) was employed to estimate population relatedness through pairwise F_ST_, and the results were visually presented using a heatmap generated by the R package ggplot2 (Wickham, 2016).

To further visualize relationships, a Neighbour-Joining tree, based on pairwise Reynolds’ genetic distances, computed with Arlequin ver. 3.5.2.2 (Excoffier and Lischer, 2010), was created using SplitsTree4 ver. 4.14.8 (Huson and Bryant, 2006). Patterns of ancestry and admixture were explored using the ADMIXTURE software v1.3. (Alexander et al., 2009), with the unsupervised model-based clustering algorithm, which estimates the individual ancestry proportions given a K number of ancestral populations. The most likely number of clusters was estimated following the cross-validation procedure, estimating the prediction errors for each K value. Circle plots were visualized using the *membercoef. Circos* function in the R package BITE ver. 1.2.0008 (Milanesi et al., 2017) with an unsupervised model-based clustering algorithm.

## 3 Results

### 3.1 Genetic diversity and ROH analyses

To evaluate the variability in Tunisian Barbarine breed, genetic diversity indices were computed (Table 1).

**TABLE 1:** Genetic diversity indices and runs of homozygosity parameters for Tunisian Barbarine sheep.

H_O_ ± sd	H_E_ ± sd	F_IS_ ± sd	F_ROH_ ± sd	MAF ± sd	Ne	N_ROH_ ± sd	L_ROH_ ± sd
0.390 ± 0.141	0.388 ± 0.109	−0.007 ± 0.031	0.017 ± 0.028	0.298 ± 0.124	140	13.500 ± 7.649	1.831 ± 1.024

H_O_, observed heterozygosity; H_E_, expected heterozygosity; F_IS_, inbreeding coefficient based on observed and expected heterozygosity; F_ROH_, inbreeding coefficient based on run of homozygosity (ROH); MAF, average minor allele frequency; Ne, contemporary effective population size; N_ROH_, mean number of ROH, per individual; L_ROH_, average length of ROH, in Mb per individual; sd, standard deviation.

The results revealed a moderate level of observed and expected heterozygosity (H_O_ = 0.390 ± 0.141; H_E_ = 0.388 ± 0.109). Additionally, the value of MAF was 0.298 ± 0.124, while the genomic inbreeding coefficients showed low values (F_IS_ = −0.007 ± 0.031; F_ROH_ = 0.017 ± 0.028).

Regarding N_e_ results, a continuous decline in effective population size can be observed across generations (Supplementary Figure S1). Specifically, the N_e_ estimated for 13 generations ago was 140. Furthermore, according to NeEstimator, the contemporary effective population size is N_e_ = 577.

The ROH analysis resulted in a total of 388 ROHs in all 24 individuals.

The N_ROH_ was 13.50 ± 7.65, while NC_ROH_ reported a mean value of 14.92 ± 10.33.

67% of ROHs had a length between 1 Mb and 2 Mb. ROHs of 2–4 and 4–8 Mb had a similar incidence of 15% and 13%, respectively, while long segments (>8 Mb) represented only 5% of the total. The L_ROH_ was 1.83 ± 1.02 Mb and the LC_ROH_ was 2.58 ± 0.98 Mb.

**FIGURE 2:**
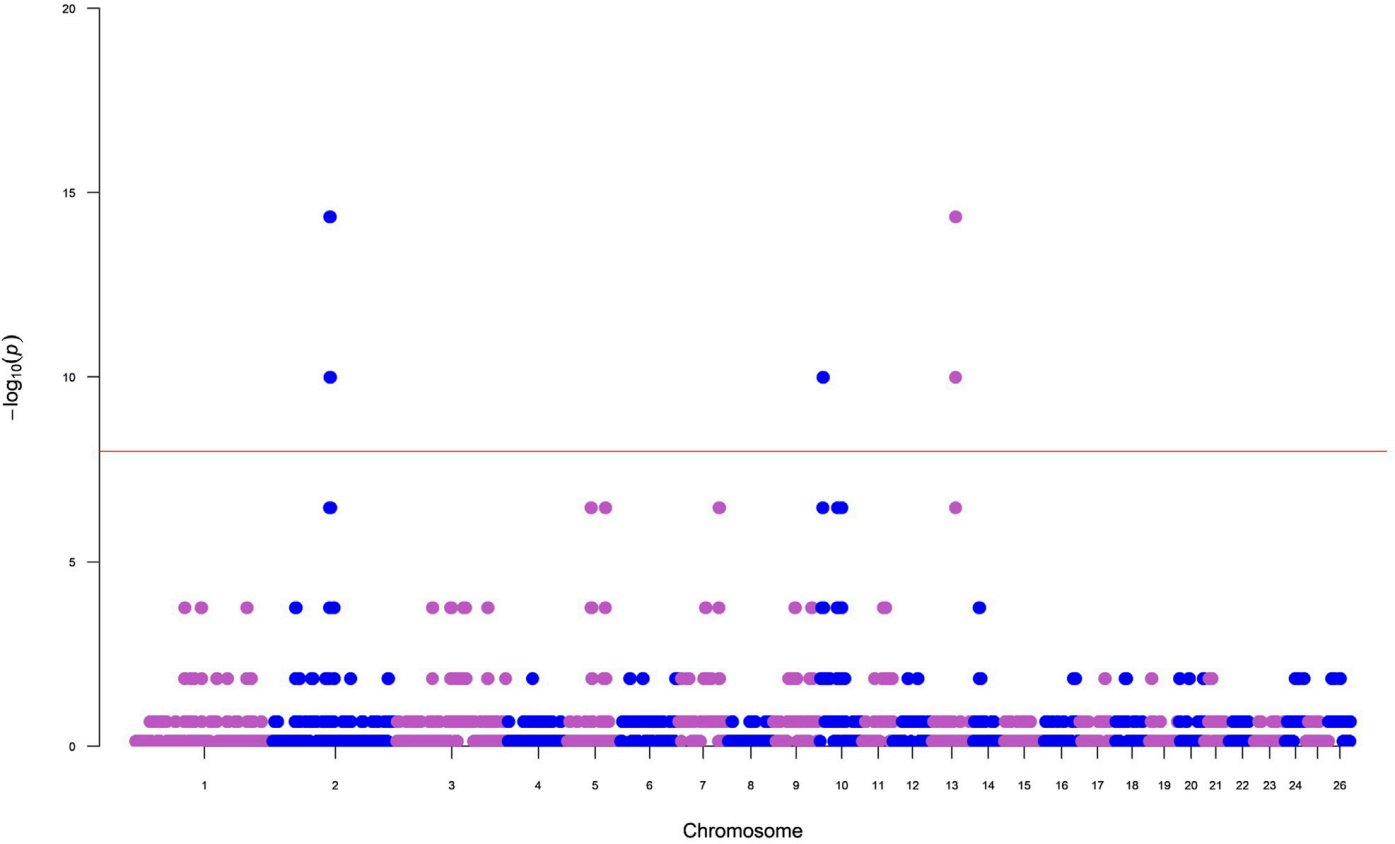
Manhattan plot for results of incidence of a single nucleotide polymorphism (SNP) in run of homozygosity (ROH) islands in Tunisian Barbarine breed.

The number of ROH per chromosome displayed a specific pattern with the highest numbers found for the first three chromosomes, consistent with expectations, as OAR1 is the longest chromosome. The top 0.1% of the SNPs-in-run percentile distribution were selected in Tunisian Barbarine breed to identify ROH islands. The results, as illustrated in a Manhattan plot (Figure 2), recognized the presence of a ROH island on OAR13, with 36 SNPs and 14 different genes, with QTLs referring to fat tail deposition and milk yield. Furthermore, a second ROH island on OAR2 with 11 SNPs and 4 genes was identified, but it does not appear to be associated with quantitative trait loci (QTLs) (Table 2).

**TABLE 2:** Runs of Homozygosity islands identified in Tunisian Barbarine breed, reporting ovine chromosome (CHR), position (start and end) and length of the island, number of harboured SNPs (N_SNP_) and annotated genes and QTLs.

CHR	Start (bp)	End (bp)	Length (bp)	N_SNP_	Genes	QTLs
13	47,169,096	49,619,573	2,450,477	36	*SHLD1; CHGB; TRMT6; MCM8; CRLS1; LRRN4; FERMT1*	Tail fat deposition
					*BMP2; LOC105609936; LOC101117437; LOC106991507*	Milk Yield
					*LOC101117953; LOC101118207; LOC101110166*	
2	114,381,876	115,516,303	1,134,427	11	*LOC105608706*	
					*LOC101106660*	
					*LOC101117569*	
					*LOC105608719*	

### 3.2 Genomic relationship and structure

**FIGURE 3:**
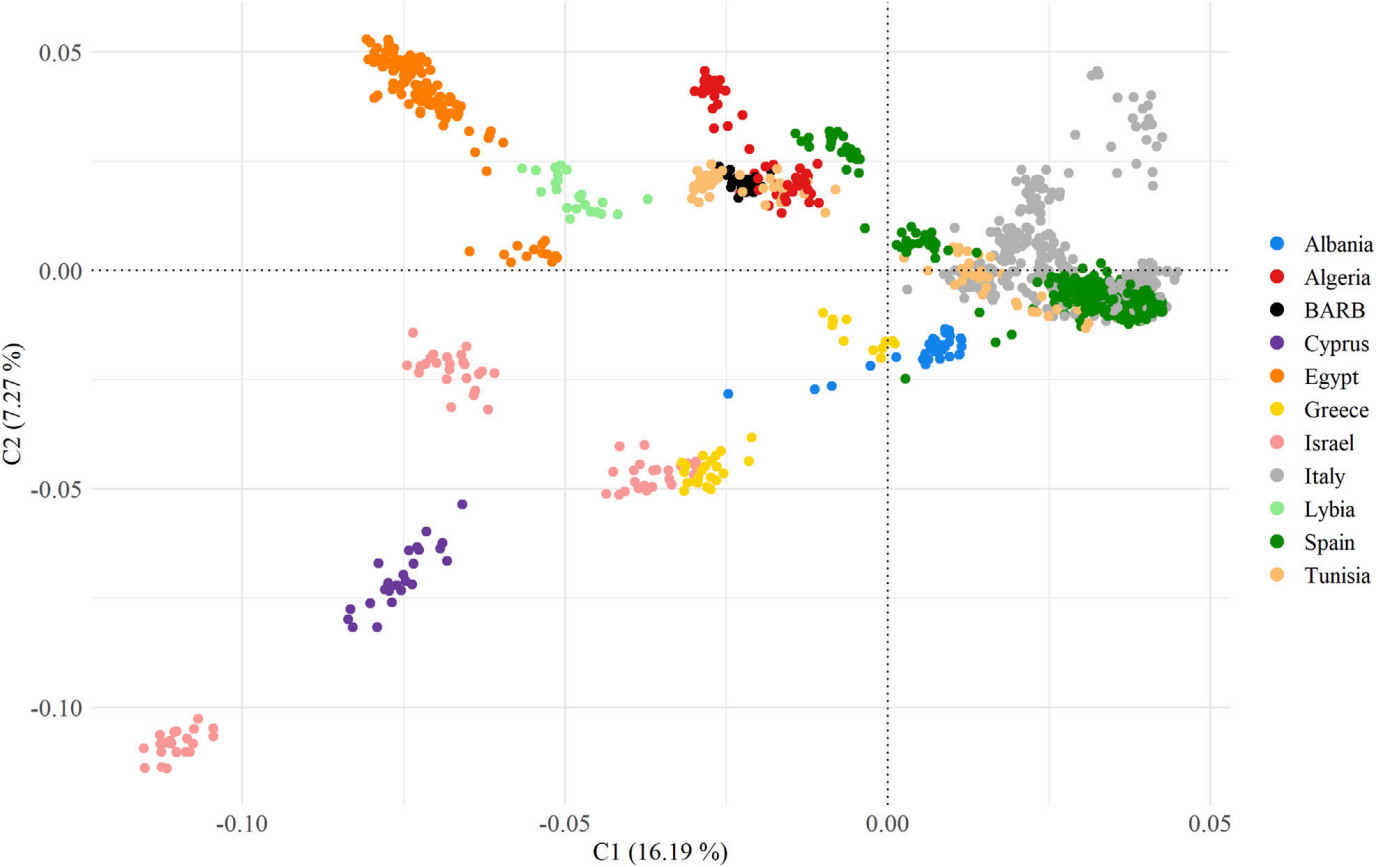
Multidimensional scaling analysis of MED_POP dataset, summarizing 60 breeds grouped according to country of origin.

To unravel the genetic relationships of the Tunisian Barbarine sheep with the Mediterranean breeds considered in the analysis, an MDS analysis based on pairwise IBS distances was performed on the MED_POP dataset (Figure 3).

The first two principal components, collectively capturing 23% of the overall variance (C1 = 16.19% and C2 = 7.27%), effectively delineate a coherent geographic distribution of the breeds.

All European breeds grouped together, giving rise to two distinct branches: the first consisting of breeds from Israel and Cyprus areas, intricately linked to a cluster comprising Greek and Albanian breeds, acting as connecting nodes to the European cluster; the second with Egyptian and Libyan (also Libyan Barbarine) breeds form a discernible cluster, closely linked to the group characterized by Tunisian Barbarine, North African (Tunisian, Algerian), and Spanish breeds.

**FIGURE 4:**
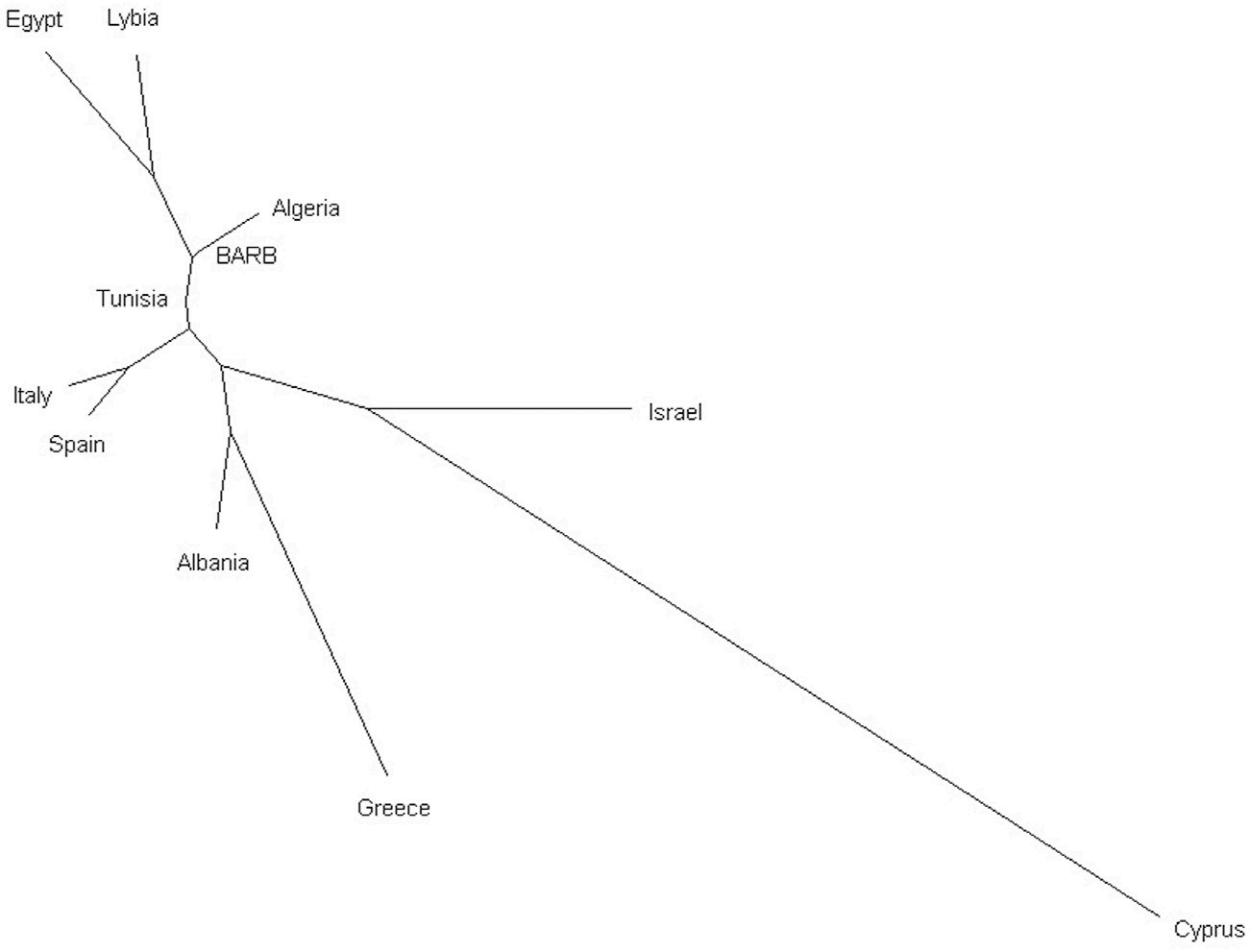
Neighbor-Joining tree based on Reynolds’ pairwise genetic distances among the 59 sheep breeds grouped by country of origin and Tunisian Barbarine.

To enhance the description of the relationship between Tunisian Barbarine and other breeds within the same cluster, a MDS plot was applied to the same dataset, with breeds grouped by names (Supplementary Figure S2). The cluster characterized primarily by the breeds from North Africa and Tunisian Barbarine, includes the Spanish Canaria de Pelo, which stands out as the outermost point in the group. Additionally, closely overlapped within this cluster are Tunisian Barbarine and Algerian Barbarine, other Tunisian breeds like D’man, Queue Fine de l’Ouest, Algerian D’men, Algerian Ouled Djellal, Rembi, Sidaoun, Hamra, Berber and Tazegzawt. The Lybian Barbarine breed does not overlap with the North African breeds or with Tunisian Barbarine. Egyptian breeds, including Farafra, Saidi, Souhagi, Ossimi and Aburamad-Halaieb-Shalateen constituted the other African cluster. Pairwise F_ST_ values calculated among all the 60 breeds ranged from 0.001 to 0.224 (Supplementary Figure S3) and identify the following pairs of breeds as the most distant: Sardinian Ancestral Black vs. Awassi (F_ST_ = 0.203), Tunisian D’man vs. Awassi (F_ST_ = 0.204) and Chios vs. Tunisian D’man (F_ST_ = 0.201). In general, Awassi obtained the highest values of F_ST_ among of the analyzed breeds. Tunisian Barbarine showed the lowest values of genetic differentiation with the following breeds: Barbarine (from a different sampling) (F_ST_ = 0.002), Algerian Barbarine (F_ST_ = 0.001), Algerian Ouled Djellal (F_ST_ = 0.004), Queue Fine de l'Ouest (F_ST_ = 0.008), Rembi, (F_ST_ = 0.003), while with the Lybian Barbarine (F_ST_ = 0.017); this higher value is also confirmed by clusters in MDS plot. Moreover, of interest are the relatively low values of F_ST_ showed by Tunisian Barbarine with Spanish Rasa Aragonesa (F_ST_ = 0.020), Algerian Sidaoun (F_ST_ = 0.021), the Egyptian Saidi (F_ST_ = 0.021) and the Italian Bagnarola (F_ST_ = 0.021). The Neighbor-Joining tree depicted in Figure 4, using Reynolds’ genetic distances, places Tunisian Barbarine in alignment with the populations of origin grouped by country, confirming the shared phylogenetic root between Tunisian Barbarine and Algeria explained by the F_ST_ results.

**FIGURE 5:**
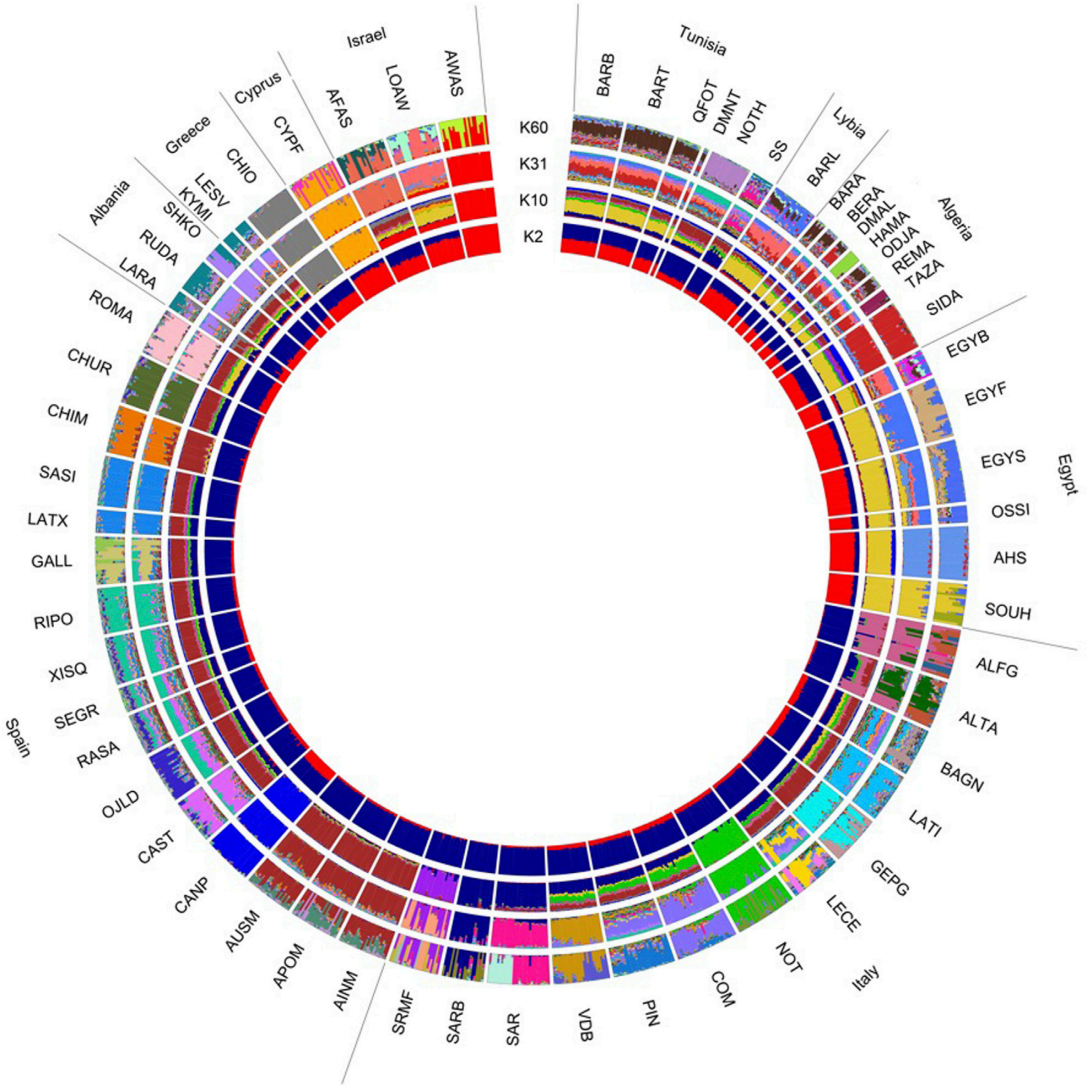
Circle plot showing ancestral clusters (K) inferred by the Admixture analysis of 60 sheep breeds.

The ADMIXTURE analysis, ranging from 2 to 60 K, provided insights into the population structure to identify ancestral components shared among different breeds (Figure 5). The breeds were clustered by geographical distribution. The results suggest that K = 31 is the most likely number of inferred populations. At K = 2, a clear separation based on geographical origin is evident, with a predominant distinction between North African breeds (including Tunisian Barbarine) from Israel and Cyprus characterized by red predominance, and European breeds (Italy, Spain, Albania, and Greece) represented by blue presence. Moving to K = 10, a shared genetic background is confirmed between Tunisian Barbarine and other North African breeds, indicating a similar internal substructure. Within the geographical regions, discernible internal substructures are also observed within breeds from Italy and Albania. Other geographical clusters (such as North African breeds) demonstrate a dominance of yellow, pattern also visible in Tunisian Barbarine, and a dominance of brown, which characterizes Spanish breeds. The internal substructure of Tunisian Barbarine and the other North African breeds is confirmed at K = 60, despite each population tending to distinguish with its own cluster. Noteworthy, besides Tunisian Barbarine, there are many other breeds exhibiting internal substructure include Awassi, Souhagi, Altamurana, Leccese, Noticiana, Sardinian Muflon, Queue fine de l’Ouest, Algerian Barbarine, Algerian Ouled Djellal and Rembi.

## 4 Discussion

### 4.1 Genetic diversity indices and ROH pattern

The study aimed to unravel the genetic structure of the Tunisian Barbarine sheep breed through comprehensive genomic analyses. Genetic diversity, which indicates the extent of genetic differences within a population, was a crucial aspect examined in this research. Actively observing and preserving this genetic diversity is essential to ensure the sustained viability of traditional breeding methods in the future, as emphasized by Asmare et al. (2023). The investigation provided valuable insights into the genetic diversity of the Tunisian Barbarine breed and its relationships with other sheep breeds. These findings shed light on population dynamics and their potential implications for breeding programs.

Notably, the observed heterozygosity (H_O_) and expected heterozygosity (H_E_) in Barbarine indicated a moderate level of genetic diversity. The differences between H_O_ and H_E_ in BARB dataset, even if low, can be attributed to several factors. Firstly, the sampling activity was contingent upon the participation of breeders within the project, thereby influencing the availability of animals for sampling. Additionally, each breeder typically maintained a small flock comprising a limited number of breeding sheep, breeding rams and their offspring. To mitigate the risk of inbreeding, only the breeding sheep and rams were included in the sampling process. Consequently, these factors collectively contributed to the relatively small size of our sample and may have influenced the observed results of genetic diversity within the Tunisian Barbarine sheep sample analysed. In comparison to previous studies on Tunisian Barbarine sheep, our results revealed higher diversity estimates than those observed in another Barbarine sample (Ahbara et al., 2022) (H_E_ = 0.301 ± 0.015 and H_O_ = 0.303 ± 0.015), whereas Bedhiaf-Romdhani et al. (2020), showed mean values of genetic diversity indices similar to the present work. This consistency across studies underscores the robustness of these findings and contributes to a comprehensive understanding of the genetic diversity within the Tunisian Barbarine population.

The present study’s results align with those of an analysis on Algerian Barbarine and seven local Algerian sheep breeds (D’men, Hamra, Ouled-Djellal, Rembi, Sidaoun, Tazegzawt, Berber): the individual H_O_ ranged from 0.220 to 0.370, with an average of 0.350, while the average H_E_ was 0.370 (Gaouar et al., 2017). Moreover, the findings are in line with those of other studies on Mediterranean breeds and southern and western European breeds reported by other authors (Kijas et al., 2012; Ciani et al., 2014). The observed values of H_E_ in Tunisian Barbarine sheep highlight a potential effect of genetic dilution due to gene flow with other thin-tailed breeds as already reported in previous studies (Gaouar et al., 2017; Bedhiaf-Romdhani et al., 2020; Ahbara et al., 2022); the crossbreeding tendencies resulted as a consequence of a shift in preference and the economic value of tail fat (Bedhiaf-Romdhani et al., 2008; Bedhiaf-Romdhani et al., 2020).

Regarding the F_IS_ results, Tunisian Barbarine showed a negative value indicating a low inbreeding. Similar findings were obtained in other studies conducted on different samples of Tunisian Barbarine (F_IS_ = −0.030 ± 0.010) (Bedhiaf-Romdhani et al., 2020), Algerian and Moroccoan breeds (F_IS_ mean = −0.040, s.d. = 0.060) (Belabdi et al., 2019), and other Algerian breeds (mean F_IS_ = − 0.070) (Belabdi et al., 2019).

In examining genetic inbreeding via ROH, Tunisian Barbarine exhibited a value aligned to that observed in another sample of Barbarine sheep (0.020) (Baazaoui et al., 2021). Interestingly, this value is significantly lower than the F_ROH_ identified by Ahbara et al. (2022) in different Tunisian Barbarine samples. Moreover, it remains lower than the values recorded for Queue Fine de l’Ouest and Noir de Thibar (Baazaoui et al., 2021).

The effective population size (N_e_) computed using SNeP v1.1 software (Barbato et al., 2015), showed a value of 140 at the 13th generation, surpassing the minimum acceptable N_e_ threshold of 100, crucial for population conservation (Mastrangelo et al., 2017; Persichilli et al., 2021).

This result was higher than that observed in Noticiana (N_e_ = 76) and lower than those observed in various Mediterranean sheep breeds like Comisana, Leccese, Sardinian Ancestral Black and Altamurana (N_e_ = 571, N_e_ = 512, N_e_ = 303; N_e_ = 224 respectively) (Ciani et al., 2014; Chessari et al., 2023).

This decline in Ne over consecutive generations underscores potential challenges on application of efficient mating plans and the need of implementation of effective conservation strategies on small local populations. It is important to note that this decline could also be influenced by sampling constraints. Sampling strategy prioritized adult animals to mitigate inbreeding, primarily due to project limitations and limited collaboration among breeders.

However, the result obtained from NeEstimator (Do et al., 2014) indicated a higher value compared to that observed in another Barbarine sample (N_e_ = 376) but lower than that observed in Noir de Thibar (N_e_ = 706) (Baazaoui et al., 2021). The analyses suggest that the genetic diversity observed in the Tunisian Barbarine breed, despite its endangered status and small population size, does not indicate significant inbreeding.

The genetic diversity indices and N_e_ values support this observation, pointing towards a relatively preserved genetic diversity within the breed. Notably, the differences between N_e_ values depend on the two different approaches used. While SNeP (Barbato et al., 2015) focuses on estimating historical Ne trends, providing insights into the genetic diversity over time, NeEstimator (Do et al., 2014) evaluates contemporary unbiased N_e_, offering a more comprehensive perspective on the genetic diversity conservation of the breed.

The evidence strongly supports the conclusion that historical crossbreeding has played a significant role in maintaining genetic diversity in Tunisian Barbarine. This is consistent with its breeding history, which involves interactions with various other breeds over time (Bedhiaf-Romdhani et al., 2008), and with results from ROH analysis.

The method based on ROH is considered one of the most powerful approaches to estimate genomic inbreeding in the livestock species, and in particular for local populations (Addo et al., 2021).

Long ROH segments typically indicate recent inbreeding events, whereas short ROH segments are associated to ancient inbreeding, due to the increased likelihood of recombination events over successive generations (Selli et al., 2021). In Tunisian Barbarine, results of L_ROH_ and LC_ROH_, lower than 3 Mb, with a 67% showing ROH length lower than 2 Mb, could suggest that inbreeding events occurred not recently, even if the count of ROH resulted quite high (388) (McQuillan et al., 2008; Kirin et al., 2010; Purfield et al., 2017). Different findings emerged among sheep breeds in Northwest Africa, including another sample of Tunisian Barbarine, Tazegzawth, Algerian Hamra, Sidaoun, and Moroccan D’Man, revealing variations in higher number and lower average length of ROH (Belabdi et al., 2019). Tazegzawth and the Tunisian Barbarine showed several long ROH fragments, in accordance with high values of F_ROH_ (i.e. 0.220 and 0.340) (Belabdi et al., 2019). In contrast, other studies reported over 45% of detected ROH falling within the 1 to 2 Mb range or a prevalence of ROH in the shortest length category (Mastrangelo et al., 2018; Getachew et al., 2020; Selli et al., 2021). It is worth noting that the utilization of low-density SNP array for ROH detection may lead to an overestimation of ROH shorter than 4 Mb (Ferenčaković et al., 2013). Despite this, Tunisian Barbarine breed exhibited a considerable number of ROH when compared to counts observed in other breeds (Selli et al., 2021).

### 4.2 Genomic relationship and structure

In conducting a comprehensive analysis of the genomic relationship and structure between Tunisian Barbarine and Mediterranean sheep populations, various statistical approaches were employed. Multidimensional scaling analysis (MDS) and population differentiation measurement, along with a Neighbor Network, confirmed findings from previous studies (Bedhiaf-Romdhani et al., 2020; Baazaoui et al., 2021). Tunisian Barbarine stands out for its remarkably low genetic differentiation, reflected in the lowest F_ST_ values, especially when compared to Algerian Barbarine, Algerian Ouled Djellal, Queue Fine de l’Ouest, and Rembi. These breeds overlap significantly in the same cluster on the MDS plot (Supplementary Figure S2). The close genetic proximity between Tunisian Barbarine and Algerian Barbarine aligns with the geographical proximity of the two countries. This closeness is further supported by the observed genetic similarity between Algerian Barbarine and Ouled Djellal, suggesting potential dilution of genetic identity due to crossbreeding with thin-tailed Ouled-Djellal sheep. The decline in the Barbarine population in Algeria by 60% between 1990 and 2000 underscores this influence (Laaziz, 2005; Gaouar et al., 2017).

The F_ST_ value between Tunisian Barbarine and Rembi is consistent with the results observed for Algerian Barbarine (F_ST_ = 0.030), supporting the possibility of crossbreeding with Rembi, another thin-tail breed (Gaouar et al., 2017). Genetic closeness between Tunisian Barbarine and Queue Fine de l’Ouest is substantiated by multiple studies, attributing the size reduction in the Tunisian Barbarine population to extensive crossbreeding between these two breeds (Bedhiaf-Romdhani et al., 2020; Baazaoui et al., 2021). The transition from fat-tailed Tunisian Barbarine sheep to thinner-tailed varieties primarily stems from preferences of butchers. Challenges in marketing fat from Tunisian Barbarine tail carcasses led to a decline in the Tunisian Barbarine population, impacting income dynamics. Additionally, the uncontrolled reproduction management and the absence of conservation programs further contribute to this scenario (Khaldi et al., 2011; Baazaoui et al., 2021). The highest F_ST_ value observed between Tunisian Barbarine and Awassi supports the notion that Awassi has not been used as a thin-tailed breed in the crossbreeding of Tunisian Barbarine. This differs from observations in Ethiopian fat-tailed sheep breeds and Awassi crossbreeding (F_ST_ = 0.004) (Getachew et al., 2020). In contrast, the F_ST_ value between Tunisian Barbarine and Libyan Barbarine (0.090) is consistent with the MDS plot, indicating relative closeness but distinct clusters. Higher F_ST_ value between Tunisian Barbarine and Noire de Thibar (F_ST_ = 0.070) suggests that, despite being a thin-tailed breed present in Tunisia (Baazaoui et al., 2021), Noire de Thibar did not significantly contribute to Barbarine’s dilution, unlike other North African breeds. In terms of genomic relationships, it can be inferred that, in the Mediterranean context, North African breeds, particularly Algerian breeds with a thin-tailed phenotype, are genetically closest and have been involved in crossbreeding plans. The results of the admixture analysis provided valuable insights into the genetic structure of these populations, and corroborated the patterns observed in the MDS plot and the F_ST_ values. Specifically, the connection through clusters between Tunisian Barbarine and Queue fine de l'Ouest, within the broader African context, was confirmed. This genetic association was consistent across various K values, with K = 10 revealing a shared ancestral component with the African cluster, characterized by a prevalence of yellow. The close genetic relationship persisted as K increased to 60, where each population formed its distinct cluster, emphasizing the robustness of the connection between Tunisian Barbarine and Queue fine de l’Ouest within the African breeds. Some Italian breeds, as already identified by other authors (Ciani et al., 2014; Chessari et al., 2023), reflect their genetic originality with homogeneous clusters.

### 4.3 ROH islands

The analysis conducted on Tunisian Barbarine enabled an exploration of its ROH pattern, revealing homozygosity hotspots associated with candidate genes and QTLs primarily linked to fat tail deposition and milk yield. The distribution of ROH on chromosomes showed a notable concentration on OAR2 and OAR3 (10%), aligning with expectations given that OAR2 is the second longest chromosome (Nosrati et al., 2021). One specific region on OAR2 (114.38–115.51 Mb) exhibited 11 SNPs and 4 genes (Table 2), with functions not corroborated in the literature. On OAR13 one island was discovered (47.16–49.61 Mb) containing 36 SNPs, 14 genes and 6 QTL related to milk yield and fat tail deposition (Table 2).

The genes *LOC101117953, LOC101118207, LOC101110166*, and the SNPs (rs412097174, rs417470080, rs422598859) were identified by Gerlando et al. (2019) in a genome-wide study on the Valle del Belice breed and annotated among those potentially influencing milk production. Regarding tail fat deposition QTL, several genes found in the ROH island in Tunisian Barbarine were identified as candidates for fat deposition.

This signal on OAR13 (47.16–49.61 Mb) is shared by other breeds, including Libyan Barbarine, Cyprus Fat-Tail, Chios, Ossimi, Italian Laticauda, and Chinese breeds, indicating a conserved fat-tail signature (Wei et al., 2015; Yuan et al., 2017; Mastrangelo et al., 2018; Baazaoui et al., 2024).

Within this region, the bone morphogenetic protein 2 (*BMP2*) gene was identified as a candidate gene for fat tail deposition by other authors (Yuan et al., 2017; Baazaoui et al., 2024) and seems to be involved in bone morphology and body shape development (Kijas et al., 2012). This is particularly intriguing given the importance of fat deposits as crucial components of adaptative physiology to survive extreme environments and conditions such as prolonged droughts, cold, and food scarcity (Ahbara et al., 2019).

Long-tailed sheep, which typically have more caudal vertebrae, tend to accumulate more fat, however it is not yet clear whether *BMP2* plays a direct role in tail fat deposition or indirectly influences fat accumulation by regulating the number of caudal vertebrae in sheep (Lu et al., 2020).

The *BMP2* gene’s impact on body size and muscle development has also been reported in cattle (Ghoreishifar et al., 2020). Additionally, *LOC101117953*, present in Tunisian Barbarine’s OAR13 island, is a newly identified gene copy resulting from a retro-transposable event originating from the protein phosphatase 1 catalytic subunit gamma (*PPP1CC*) gene, also located on ovine chromosome 13 (Pan et al., 2022). These findings underscore the importance of identifying unique traits in response to biodiversity loss. Despite genetic dilution with other thin tailed breeds, Tunisian Barbarine sheep still exhibits physiological mechanisms related to fat storage, typical of fat-tailed sheep breeds that inhabit tropical environments. This mechanism of storing and mobilizing body reserves is potentially advantageous in coping with heat stress and limited food and forage availability (Atti et al., 2004).

## 5 Conclusion

This study delved into the genetic structure of the Tunisian Barbarine sheep breed, revealing insights into its diversity and relationships with other breeds from the Mediterranean area. The results also confirmed the genetic dilution trend of Tunisian Barbarine towards thin-tailed breeds, as previously observed by other authors studying different Barbarine samples from various regions. The genetic diversity indices showed a moderate level of genetic diversity in Tunisian Barbarine and low inbreeding. ROH analysis identified ROH islands linked to important distinctive traits such as the fat tail and its metabolism. The genomic relationship analysis underscored the close genetic proximity between Tunisian Barbarine and related thin-tailed breeds such as Queue fine de l’Ouest or Algerian Ouled Djellal, suggesting potential crossbreeding impacts. The admixture analysis unveiled distinct genetic patterns, emphasizing Tunisian Barbarine’s unique identity within the Mediterranean context and its genetic closeness with the African breeds. In summary, despite the observed genetic dilution in the Tunisian Barbarine breed due to crossbreeding with thin-tailed sheep breeds from Africa, the identification of ROH islands on chromosome 13 offers essential insights for devising conservation programs that recognize and appreciate the distinctive traits of the breed. This genomic region has shown the potential of tail fat deposition, emphasizing possibly adaptation mechanism to environmental selection pressures. This finding confirms the importance of preserving the unique genetic characteristics of local breeds in light of ongoing crossbreeding practices and future environmental challenges.

## Data Availability

The original contributions presented in the study are publicly available. This data can be found here: https://figshare.com/s/110c7181af58820b4ab4.

## Supplementary material

SUPPLEMENTARY FIGURE S1 Trends in historic effective population size (Ne) for Tunisian Barbarine breed.
SUPPLEMENTARY FIGURE S2
Multidimensional scaling analysis of MED_POP dataset, comprehensive of 60 breeds in total, grouped according to breeds’ name. Tunisian Barbarine (BARB) in black.
SUPPLEMENTARY FIGURE S3
Pairwise FST values estimated between Barbarine and MED_POP.
