# Supplementary material for: Insight into the current genomic diversity, conservation status and population structure of Tunisian Barbarine sheep breed
Source: Front Genet. 2024 May 31;15:1379086. doi: 10.3389/fgene.2024.1379086 (PMC11176520; doi:10.3389/fgene.2024.1379086)
Supplement: Supplementary file 1 [file Table1.docx]

**Supplementary Table 1.** Breeds, acronyms (code) and number of individuals (NID) involved in the analyses explicated for all the datasets after quality control and references.

| Breed | N° ID | Code | Tail type | Origin | References |
| --- | --- | --- | --- | --- | --- |
| Lara | 9 | LARA | thin | **Albania** | (20) |
| Ruda | 16 | RUDA | thin | **Albania** | (20) |
| Shkodrane | 10 | SHKO | thin | **Albania** | (20) |
| Algerian Barbarine | 5 | BARA | thin | **Algeria** | (21) |
| Berber | 6 | BERA | fat | **Algeria** | (21) |
| Algerian D'men | 5 | DMAL | thin | **Algeria** | (21) |
| Hamra | 6 | HAMA | thin | **Algeria** | (21) |
| Algerian Ouled Djellal | 6 | ODJA | thin | **Algeria** | (21) |
| Rembi | 6 | REMA | thin | **Algeria** | (21) |
| Sidaoun | 24 | SIDA | thin | **Algeria** | (21) |
| Tazegzawt | 6 | TAZA | thin | **Algeria** | (21) |
| Cyprus Fat Tail | 24 | CYPF | fat | **Cyprus** | (22) |
| Aburamad-Halaieb-Shalateen | 26 | AHS | fat | **Egypt** | (24) |
| Egyptian Barki | 13 | EGYB | fat | **Egypt** | (22) |
| Farafra | 29 | EGYF | fat | **Egypt** | (24) |
| Saidi | 30 | EGYS | fat | **Egypt** | (24) |
| Ossimi | 8 | OSSI | fat | **Egypt** | (23) |
| Souhagi | 20 | SOUH | fat | **Egypt** | (24) |
| Chios | 23 | CHIO | semi-fat | **Greece** | (22) |
| Kymi | 6 | KYMI | semi-fat | **Greece** | (20) |
| Lesvos | 6 | LESV | semi-fat | **Greece** | (20) |
| Afec Assaf | 24 | AFAS | fat | **Israel** | (22) |
| Improved Awassi | 23 | AWAS | fat | **Israel** | (22) |
| Local Awassi | 24 | LOAW | fat | **Israel** | (22) |
| AltamuranaFG | 24 | ALFG | thin | **Italy** | (25) |
| Altamurana | 23 | ALTA | thin | **Italy** | (25) |
| Bagnolese | 23 | BAGN | thin | **Italy** | (25) |
| Comisana | 30 | COM | thin | **Italy** | (27) |
| GentilePuglia | 24 | GEPG | fat | **Italy** | (25) |
| Laticauda | 24 | LATI | thin | **Italy** | (25) |
| Leccese | 25 | LECE | thin | **Italy** | (25) |
| Noticiana | 30 | NOT | thin | **Italy** | (28) |
| Pinzirita | 30 | PIN | thin | **Italy** | (27) |
| Sarda | 30 | SAR | thin | **Italy** | (27) |
| Sardinian Ancestral Black | 20 | SARB | thin | **Italy** | (25) |
| Sardinian Mouflon | 24 | SRMF | thin | **Italy** | (26) |
| Valle Del Belice | 27 | VDB | thin | **Italy** | (27) |
| Lybian Barbarine | 23 | BARL | fat | **Lybia** | (23) |
| Australian Industry Merino | 24 | AINM | thin | **Spain** | (22) |
| Australian Poll Merino | 24 | APOM | thin | **Spain** | (22) |
| Australian Merino | 24 | AUSM | thin | **Spain** | (22) |
| Canaria de pelo | 24 | CANP | thin | **Spain** | (29) |
| Castellana | 22 | CAST | thin | **Spain** | (22) |
| Chinese Merino | 23 | CHIM | thin | **Spain** | (22) |
| Churra | 24 | CHUR | thin | **Spain** | (22) |
| Gallega | 23 | GALL | thin | **Spain** | (29) |
| Latxa | 11 | LATX | thin | **Spain** | (20) |
| Ojalada | 24 | OJLD | thin | **Spain** | (22) |
| Rasa Aragonesa | 20 | RASA | thin | **Spain** | (22) |
| Ripollesa | 22 | RIPO | fat | **Spain** | (20) |
| Roja Mallorquina | 23 | ROMA | thin | **Spain** | (29) |
| Sasi Ardi | 24 | SASI | thin | **Spain** | (20) |
| Segureña | 12 | SEGR | thin | **Spain** | (20) |
| Xisqueta | 23 | XISQ | thin | **Spain** | (20) |
| Tunisian Barbarine | 23 | BART | thin | **Tunisia** | (30) |
| Tunisian D'man | 2 | DMNT | thin | **Tunisia** | (30) |
| Noire de Thibar | 20 | NOTH | thin | **Tunisia** | (30) |
| Queue fine de l'Ouest | 12 | QFOT | thin | **Tunisia** | (30) |
| Sicilio-Sarde | 11 | SS | fat | **Tunisia** | (30) |
| Tunisian Barbarine | 24 | **BARB** | fat | **Tunisia** | **This study** |
