# Supplementary figures and images for: Insight into the current genomic diversity, conservation status and population structure of Tunisian Barbarine sheep breed
Source: Front Genet. 2024 May 31;15:1379086. doi: 10.3389/fgene.2024.1379086 (PMC11176520; doi:10.3389/fgene.2024.1379086)

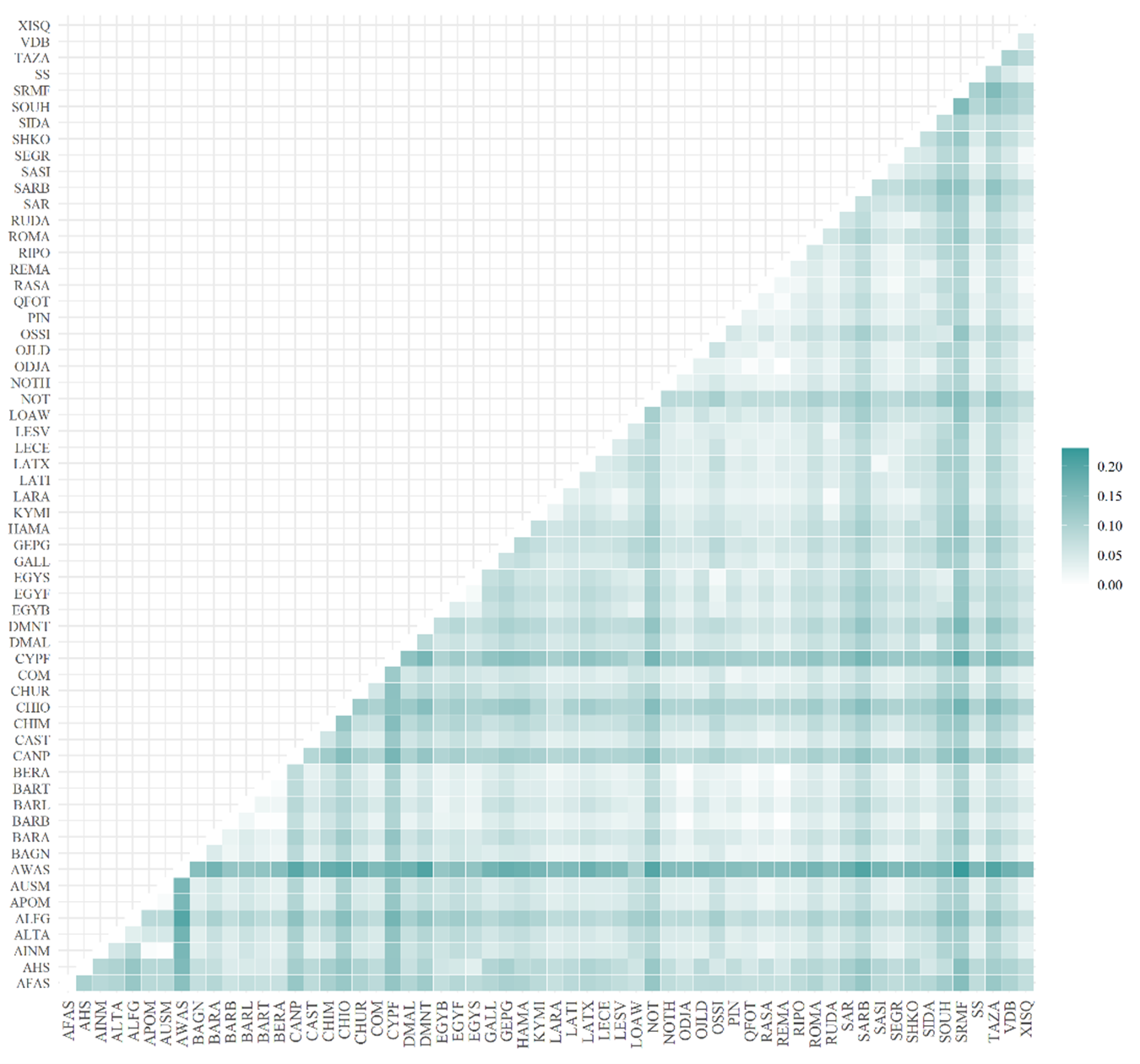

Supplement: Supplementary file 2 [file Image3.tif]

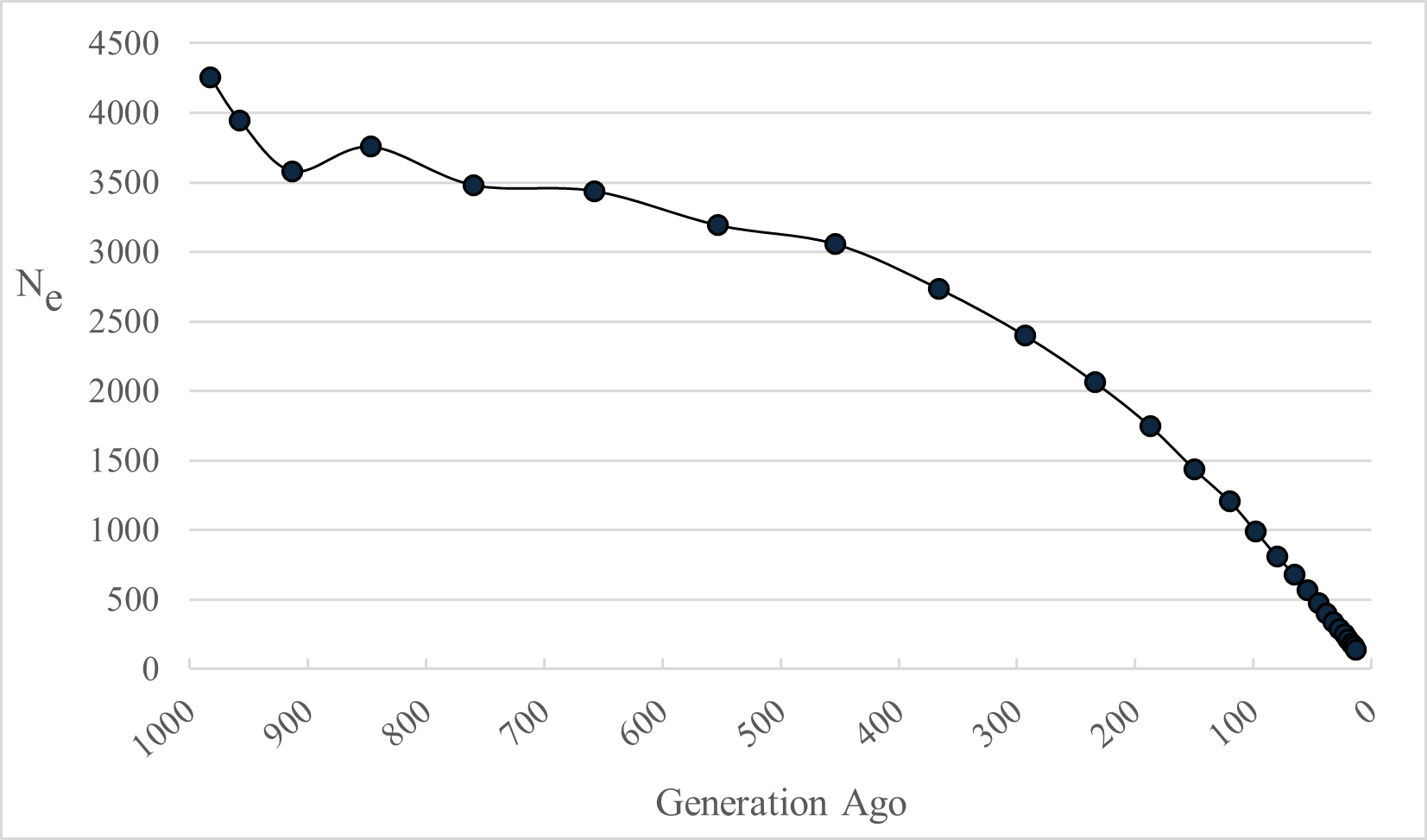

Supplement: Supplementary file 3 [file Image1.tif]
