# Supplementary material for: Insight into the current genomic diversity, conservation status and population structure of Tunisian Barbarine sheep breed
Source: Front Genet. 2024 May 31;15:1379086. doi: 10.3389/fgene.2024.1379086 (PMC11176520; doi:10.3389/fgene.2024.1379086)
Supplement: Supplementary file 4 [file Image2.pdf]

Supplementary Figure 2. Multidimensional scaling analysis of MED_POP dataset, comprehensive of 60 breeds in total, grouped according to breeds’ name. Tunisian Barbarine (BARB) in black.

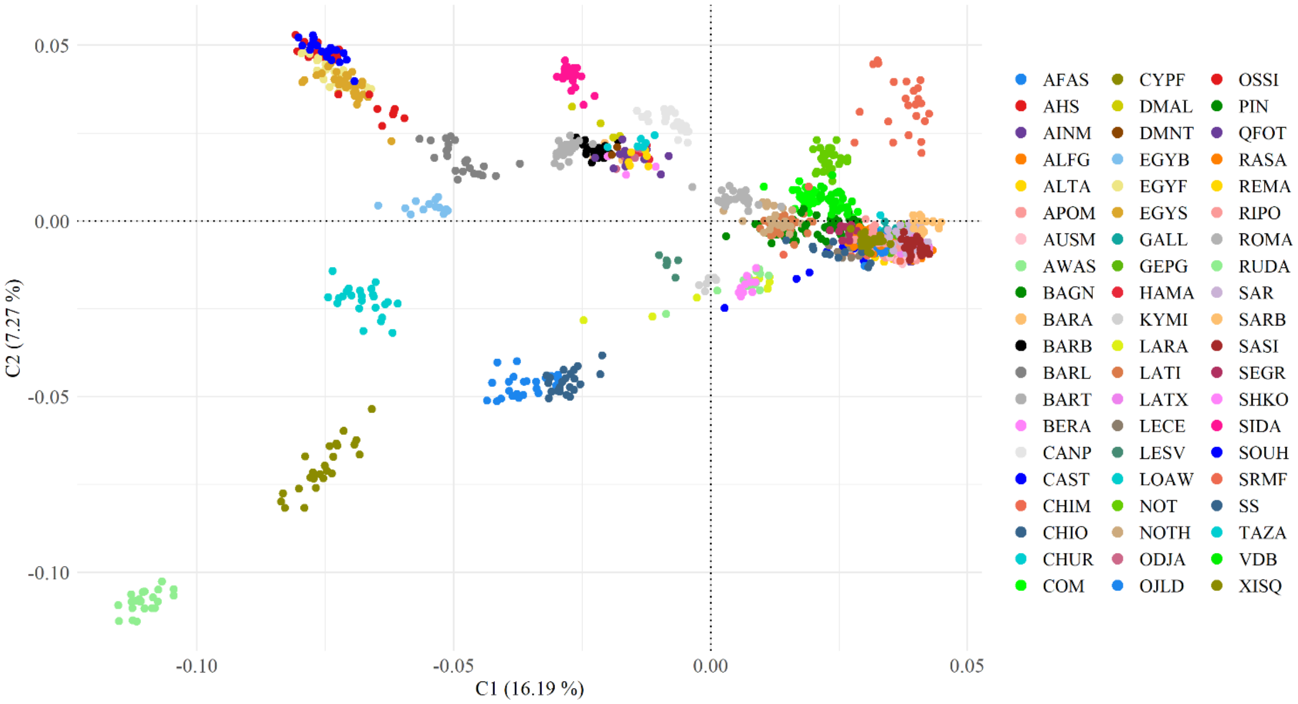

Assaf (AFAS); Aburamad-Halaieb-Shalateen (AHS); Australian Industry Merino (AINM); AltamuranaFG (ALFG); Altamurana (ALTA); Australian Poll Merino (APOM); Australian Merino (AUSM); Improved Awassi (AWAS); Bagnolese (BAGN); Algerian Barbarine (BARA); Barbarine (BARB)*; Lybian Barbarine (BARL); Tunisian Barbarine (BART); Berber (BERA); Canaria de pelo (CANP); Castellana (CAST); Chinese Merino (CHIM); Chios (CHIO); Churra (CHUR); Comisana (COM); Cyprus Fat Tail (CYPF); Algerian D'men (DMAL); Tunisian D'man (DMNT); Egyptian Barki (EGYB); Farafra (EGYF); Saidi (EGYS); Gallega (GALL); GentilePuglia (GEPG); Hamra (HAMA); Kymi (KYMI); Lara (LARA); Laticauda (LATI); Latxa (LATX); Leccese (LECE); Lesvos (LESV); Local Awassi (LOAW); Noticiana (NOT); Noire de Thibar (NOTH); Algerian Ouled Djellal (ODJA); Ojalada (OJLD); Ossimi (OSSI); Pinzirita (PIN); Queue fine de l'Ouest (QFOT); Rasa Aragonesa (RASA); Rembi (REMA); Ripollesa (RIPO); Roja Mallorquina (ROMA); Ruda (RUDA); Sarda (SAR); SardinianAncestralBlack (SARB); Sasi Ardi (SASI); Segureña (SEGR); Shkodrane (SHKO); Sidaoun (SIDA); Souhagi (SOUH); Sardinian Mouflon (SRMF); Sicilio-Sarde (SS); Tazegzawt (TAZA); Valle Del Belice (VDB); Xisqueta (XISQ).
